# In vivo assessment of catheter positioning accuracy and prolonged irradiation time on liver tolerance dose after single-fraction ^192^Ir high-dose-rate brachytherapy

**DOI:** 10.1186/1748-717X-6-107

**Published:** 2011-09-05

**Authors:** Lutz Lüdemann, Christian Wybranski, Max Seidensticker, Konrad Mohnike, Siegfried Kropf, Peter Wust, Jens Ricke

**Affiliations:** 1 Department of Radiation Therapy, Charité Medical Center, Berlin, Germany; 2 Department of Radiology and Nuclear Medicine, Otto von Guericke University, Magdeburg, Germany; 3 Department of Biometrics and Medical Informatics, Otto von Guericke University, Magdeburg, Germany

## Abstract

**Background:**

To assess brachytherapy catheter positioning accuracy and to evaluate the effects of prolonged irradiation time on the tolerance dose of normal liver parenchyma following single-fraction irradiation with ^192 ^Ir.

**Materials and methods:**

Fifty patients with 76 malignant liver tumors treated by computed tomography (CT)-guided high-dose-rate brachytherapy (HDR-BT) were included in the study. The prescribed radiation dose was delivered by 1 - 11 catheters with exposure times in the range of 844 - 4432 seconds. Magnetic resonance imaging (MRI) datasets for assessing irradiation effects on normal liver tissue, edema, and hepatocyte dysfunction, obtained 6 and 12 weeks after HDR-BT, were merged with 3D dosimetry data. The isodose of the treatment plan covering the same volume as the irradiation effect was taken as a surrogate for the liver tissue tolerance dose. Catheter positioning accuracy was assessed by calculating the shift between the 3D center coordinates of the irradiation effect volume and the tolerance dose volume for 38 irradiation effects in 30 patients induced by catheters implanted in nearly parallel arrangement. Effects of prolonged irradiation were assessed in areas where the irradiation effect volume and tolerance dose volume did not overlap (mismatch areas) by using a catheter contribution index. This index was calculated for 48 irradiation effects induced by at least two catheters in 44 patients.

**Results:**

Positioning accuracy of the brachytherapy catheters was 5-6 mm. The orthogonal and axial shifts between the center coordinates of the irradiation effect volume and the tolerance dose volume in relation to the direction vector of catheter implantation were highly correlated and in first approximation identically in the T1-w and T2-w MRI sequences (*p *= 0.003 and *p *< 0.001, respectively), as were the shifts between 6 and 12 weeks examinations (*p *= 0.001 and *p *= 0.004, respectively). There was a significant shift of the irradiation effect towards the catheter entry site compared with the planned dose distribution (*p *< 0.005). Prolonged treatment time increases the normal tissue tolerance dose. Here, the catheter contribution indices indicated a lower tolerance dose of the liver parenchyma in areas with prolonged irradiation (*p *< 0.005).

**Conclusions:**

Positioning accuracy of brachytherapy catheters is sufficient for clinical practice. Reduced tolerance dose in areas exposed to prolonged irradiation is contradictory to results published in the current literature. Effects of prolonged dose administration on the liver tolerance dose for treatment times of up to 60 minutes per HDR-BT session are not pronounced compared to effects of positioning accuracy of the brachytherapy catheters and are therefore of minor importance in treatment planning.

## 1 Background

Single-fraction ^192^Ir high-dose-rate brachytherapy (HDR-BT) of the liver is an ablation technique which has shown promising results with respect to safety and efficacy in the treatment of nonresectable primary and secondary liver malignancies [1-3]. HDR-BT provides steep dose gradients at the surface of the target volume due to the low *γ*-ray energy of ^192^Ir and use of a point source, and thus can be used to treat several malignancies in one session or recurrent malignancies sequentially without seriously impairing the functional hepatic reserve [4]. To prevent recurrence at the tumor margins, catheter placement and dwell positions of the ^192 ^Ir point source have to be carefully planned [5]. The accuracy of dose application is predominantly dependent on catheter positioning. Computed tomography (CT) was used to monitor catheter implantation, and 3D CT datasets acquired in breath-hold were used for treatment planning. For irradiation patients were transferred from the CT unit to the brachytherapy unit. Dislocation of catheters during patient transfer might be a potential source of error with respect to correct dose application at the target site. Additionally, the liver is an elastic organ and could be deformed between catheter implantation and irradiation.

The treatment of larger tumors with an ^192^Ir point source requires the implantation of approximately 1 catheter for each 1 - 2 cm of tumor diameter. The contributions of several catheters with numerous dwell positions to the planned dose in a large part of the target volume lead to regional prolongation of irradiation. Several authors describe an increased normal tissue dose tolerance for prolonged radiation therapy or pulsed dose rate (PDR) radiation therapy [6,7] even if the total irradiation time is less than one hour [8].

The present study aims at addressing two methodical aspects of HDR-BT: First, to investigate the limits of catheter positioning accuracy and its clinical importance. Second, to investigate if effects of prolonged irradiation times on the tolerance dose of normal liver parenchyma are important for clinical practice and may have to be taken into account in treatment planning.

## 2 Methods

### Study population

In this study we retrospectively analyzed irradiation effects on normal liver tissue in 50 consecutive patients who underwent CT-guided single-fraction HDR-BT as part of a clinical phase II study prospectively assessing local tumor control. In 50 HDR-BT sessions a total of 76 solid primary or secondary liver tumors were treated (1 - 4 malignant tumors per session). The study was approved by the local ethics committee. Written informed consent was obtained from all patients.

### Interventional technique

The interventional technique has been described in detail elsewhere [9]. In brief, a T2-weighted (T2-w) respiratory-triggered ultrafast turbo spin echo (UTSE) and a T1-weighted (T1-w) breath-hold gradient echo (GRE) sequence with administration of the hepatocyte-specific contrast agent gadobenate dimeglumine (Gd-BOPTA (Multihance), Bracco, Princeton, NJ) were acquired to delineate primary and secondary liver lesions (see Follow-up section below). The brachytherapy catheters were positioned using CT guidance (Somatom 4, Siemens, Erlangen, Germany), i.e., CT scans were acquired continuously during the interventional procedure with an image reconstruction rate of 12 per second to monitor actual catheter location. They were placed in 6F angiographic sheaths (Radiofocus, Terumo, Japan), which were implanted in Seldinger technique within the tumors. The angiographic sheaths were sutured to the skin. After catheter positioning, a spiral CT scan of the liver (matrix size, 512 × 512; slice thickness, 5 mm; increment, 5 mm) enhanced by intravenous administration of iodine contrast medium (100 ml Ultravist 370; flow, 1 ml/s; start delay, 80s) was acquired in breath-hold technique for treatment planning. Four catheters were implanted on average per HDR-BT session (range, 1 - 11 catheters).

### Treatment planning and irradiation

**Figure 1:**
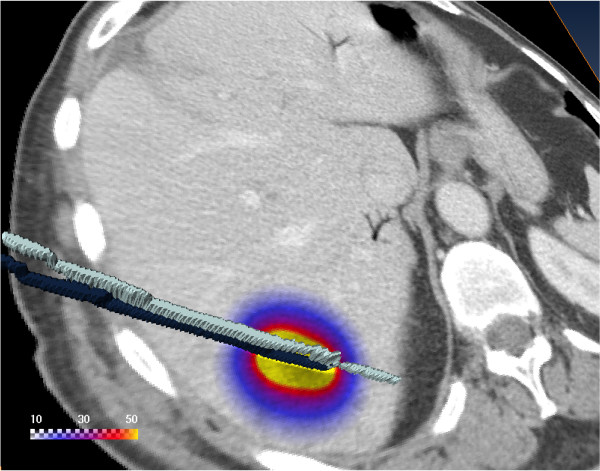
**Geometry**. The 3D visualization shows a CT slice with the calculated dose in Gy overlayed. The dose is applied using two catheters. The two catheters were visualized in 3D using surface rendering of the catheters labeled in the CT scan.

Treatment was planned using the BrachyVision software package, version 7.1 (Varian Medical Systems, Palo Alto, CA). The dwell positions and irradiation times were optimized to ensure delivery of the prescribed dose to the entire clinical target volume (CTV), see Figure 1. The 24-channel HDR afterloading system (Gammamed 12i, Varian, Charlottesville, VA) employed a ^192^Ir source (nominal source strength, 370GBq). A dose of 15, 20, or 25Gy was prescribed, which was planned to enclose the lesion (clinical target volume). Compromises were necessary if organs of risk such as the stomach, small intestine, or a large bile duct were very close to the target. No upper limit was defined for the dose within the tumor volume. To preserve liver function after irradiation, one third of the liver parenchyma should receive a dose of less than 5Gy. The effective irradiation time needed to apply the target dose with all catheters was corrected according to the actual ^192 ^Ir source strength. We usually limit the maximum irradiation time to 60 minutes to increase patient comfort. The catheters were then sequentially connected to the afterloading system according to the prescribed enumeration, and irradiation was started at the most distant dwell position in each catheter. All dwell positions within one catheter were sequentially irradiated without any delay. An interval of approx. 2 - 3 minutes was required for connecting each catheter. Manual sequential connection of the catheters was necessary because only a single adapter was available for connecting the catheters to the afterloader. The exposure times were in the range of 844 - 4432 seconds.

### Follow-up

A total of 161 MRI examinations were performed 6 ± 2 weeks and 12 ± 2 weeks after HDR-BT. The MRI protocol comprised the following sequences (Gyroscan NT Intera, Philips, The Netherlands) [10]: T2-w respiratory-triggered UTSE (echo time/repetition time (TE/TR), 90/2100 ms; echo train length (ETL), 21; slice thickness, 8 mm, acquired in interleaved mode with no gap) with fat suppression to assess the extent of interstitial edema and T1-w breath-hold GRE (TE/TR 5/30 ms; flip angle,30°; slice thickness, 8 mm, acquired in, interleaved mode with no gap) 2 h after intravenous injection of 15 ml gadobenate dimeglumine (Gd-BOPTA (Multihance), Bracco, Princeton, NJ). The hepatocyte-specific contrast agent gadobenate dimeglumine allowed visualization of the extent of hepatocyte dysfunction. The underlying mechanism of intracellular uptake is a polyspecific organic anionic transport [11-13].

### Image registration

**Figure 2:**
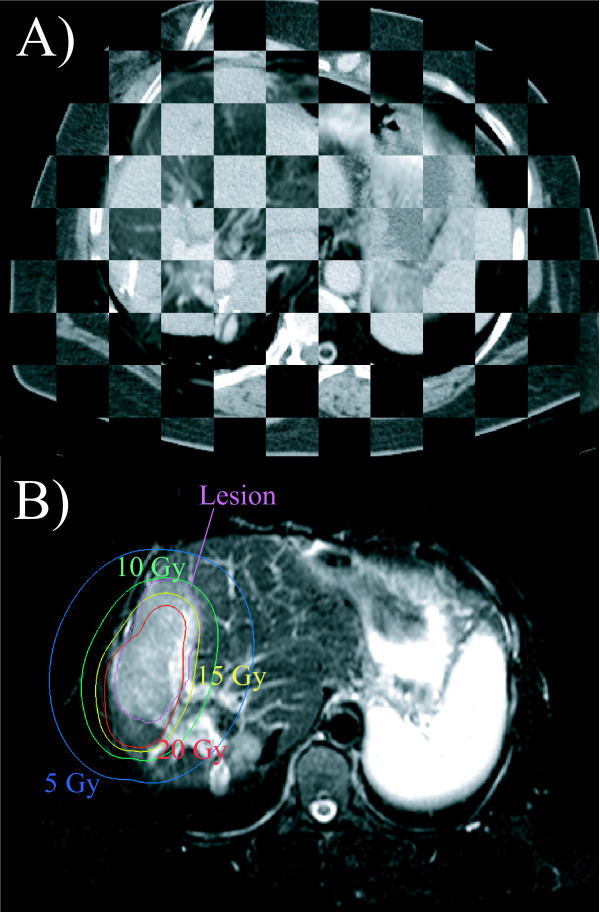
**Image registration**. A) T2-w image coregistered with the planning CT. Note that only the liver was coregistered and therefore good matching of the images was only achieved for the liver. B) T2-w image showing segmented lesion and isodoses at 12-week follow-up. A prononounced shift of the irradiation effect with respect to the planned dose distribution as shown in this example was typically not found.

Merging of the 3D dosimetry data calculated by BrachyVision with the corresponding follow-up MRI scans was accomplished using an independent image registration implementation within the 3D visualization software Amira 3.1 (Mercury Computer Systems, Berlin, Germany). The image voxel-property-based registration method allowed affine transformation (12 degrees of freedom: 3 rotations, 3 translations, 3 scalings, and 3 shears) by exploring the normalized mutual information (NMI) [14], see Figure 2A. The liver including a 1-cm margin was segmented in the treatment planning CT. The segmented data served as reference for registration to optimize registration accuracy for the liver. Registration accuracy was validated using intrahepatic vessel bifurcations as landmarks. Three to four landmarks were set in the CT and MRI image data of ten patients. Distances between the landmarks in the coregistered images (CT vs. MRI) were determined using the differences between the absolute positions determined with Amira. A total of 120 coregistered landmark combinations were evaluated.

### Calculation of normal liver tissue tolerance dose

**Figure 3:**
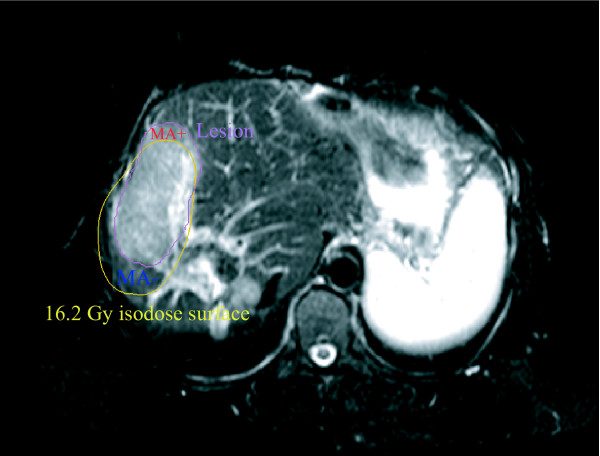
**Mismatch areas**. T2-w image showing segmented irradiation effect and 16.2Gy isodose encompassing the corresponding tolerance dose volume. A very pronounced shift of the irradiation effect with respect to the isodoses is shown to illus-trate the likely maximum inaccuracy of catheter positioning. Mismatch areas in which we observed a dose response at doses smaller than the tolerance dose of the total irradiation effect are indexed with "*MA*+" and mismatch areas in which we did not observe a dose response at doses higher than the tolerance dose of the total irradiation effect are indexed with "*MA*- ".

The borders of hyperintensity on T2-w images (interstitial edema) and hypointensity on late Gd-BOPTA-enhanced T1-w images (hepatocyte dysfuntion) around the irradiated liver tumors were outlined, see Figure 2B. The volume of each irradiation effect was determined. As the next step, we used this volume to calculate the 3D-isodose, which was confined to the liver and encompassed a corresponding volume (± 1%). The calculated isodose was taken as a surrogate for the tolerance dose of normal liver tissue assuming consistency between an observed radiation effect and the dose applied [9]. The volume encompassed by the isodose surface will be referred to as tolerance dose volume in the following. The mismatch areas between both volumes were investigated in detail for the effect of prolonged irradiation time, see Figure 3.

### Measurement of lesion volume shift in relation to planned volume

Potential inaccuracies of the treatment planning procedure or catheter dislocation were analyzed by calculating the shift between the center coordinates of the irradiation effect volume and the tolerance dose volume using the coordinate system of the planning CT. Only those brachytherapies were evaluated in which the catheters were implanted unidirectionally, i.e., in parallel (n = 38).

The direction vector of an implanted catheter was calculated from the coordinates of the catheter skin entry site and the catheter tip in the treatment planning CT. If more than one catheter was implanted, an average coordinate from the coordinates of the entry sites and of the catheter tips was calculated. The direction vector of catheter implantation was converted into a unit vector $\vec{e}$ with unit length 1 *cm*.

The shift vector $\vec{S}$ describing the shift between the irradiation effect volume and the tolerance dose volume was calculated from the center coordinates of both volumes. The scalar product of the unit vector and the shift vector, $S_{axial}=\vec{e}\cdot \vec{S}$, was taken as a measure of the shift between irradiation effect volume and tolerance dose volume axial to the direction vector of catheter implantation. It serves as a surrogate for catheter dislocation within the catheter track. The vector product of both vectors, $S_{ortho}=|\vec{e}\times \vec{S}|$, provides a measure of the orthogonal shift between the center coordinates of the irradiation effect volume and the tolerance dose volume in relation to the direction vector of catheter implantation. Since movement of the brachytherapy catheters within the liver is limited to the catheter track the orthogonal shift results mainly from methodical limitations of image registration due to local liver deformation. The vector product thus serves as an additional surrogate for registration inaccuracy.

An asymmetry coefficient of the scalar and vector product was calculated to differentiate between a systematic shift and registration inaccuracy:

(1) $$AC_{S}=\frac{|S_{axial}|-S_{ortho}}{0.5\left(|S_{axial}|+S_{ortho}\right)}$$

A positive value of the asymmetry coefficient indicates a shift predominantely parallel to the direction vector of the implanted catheter, whereas a negative value indicates a shift predominantly orthogonal to the direction vector of the implanted catheter.

### Evaluation of prolonged irradiation time

Irradiation took up to 4432 seconds (≈ 74 minutes) using multiple catheters with numerous dwell positions of the ^192^Ir source. Therefore, in areas with significant dose contribution of several catheters, dose delivery time was prolonged and may be characterized as pulsed dose administration. The effects of regionally longer, pulsed irradiation were investigated in areas where the extent of hepatocyte dysfunction and edema was not consistent with the applied dose. Only radiation effects induced by at least 2 brachytherapy catheters were assessed (n = 48).

We used a boolean tool implemented in Amira 3.1 to identify nonoverlapping areas of the irradiation effect volume and the corresponding tolerance dose isovolume (confined to the liver). These areas will be referred to as mismatch areas in the following. Mismatch areas where edema or hepatocyte dysfunction occurred at doses smaller than the tolerance dose of the total irradiation effect are indexed with '"MA+". Conversely, mismatch areas in which edema or hepatocyte dysfuntion did not manifest at doses exceeding the tolerance dose of the total irradiation effect are indexed with „MA-", see Figure 3. The '"MA+" and „MA-" mismatch areas by definition have identical volumes.

**Figure 4:**
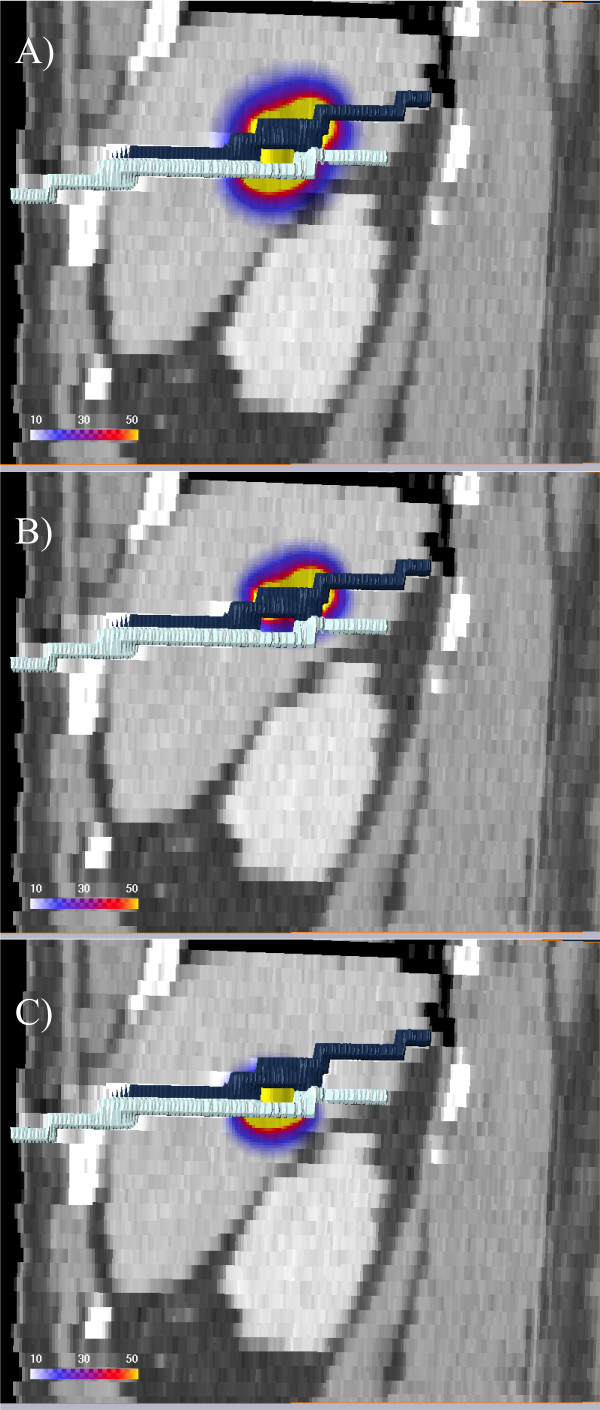
**Dose separation**. The 3D visualization shows a coronal CT reconstruction with the calculated dose in Gy overlayed using the patient in Fig. 1. The dose is applied using two catheters. The two catheters were visualized in 3D using surface rendering of the catheters labeled in the CT scan. A) Total dose, *D**_tot _***, overlayed. B) Dose applied by the cranial catheter, *D*_1_. C) Dose applied by the caudal catheter, *D*_2 _.

A comprehensive description of the time course of irradiation in brachytherapy is difficult since multiple catheters with numerous dwell positions contribute to dose fractionation in each voxel. First, the total voxel dose, *D*_*tot *_(*x*,*y*,*z*), depends on the voxel position. Second, the dose contribution of each catheter, *D_i_*(*x*, *y*, *z*), depends on the voxel position, (*x*,*y*,*z*), where *i *is the catheter number. Third, each voxel is irradiated with a different dose administration scheme, *D_tot _*(*x*,*y*,*z*) = ∑*_n _D_i_*(*x*,*y*,*z*), where *n *is the number of catheters. The BrachyVision software allows separation of the total dose map, *D_tot _*(*x*,*y*,*z*), into *n *separate dose maps, *D_i_*(*x*,*y*,*z*), for each catheter ***i***, see Figure 4. We calculated a total of 202 separate treatment plans using the treatment planning system to determine the contribution of each catheter to the total of 48 irradiation effects. To estimate the prolongation of irradiation by the ^192^Ir HDR source we calculated a catheter contribution index, *I_P_*(*x*,*y*,*z*), that uses the number of dose contribution pulses:

(2) $$|I_{P}\left(x,y,z\right)|\;=n-\overset{n}{\underset{i=1}{\sum }}\sqrt{{\left(2\cdot \frac{D_{i}\left(x,y,z\right)}{D_{tot}\left(x,y,z\right)}-1\right)}^{2}}$$

**Figure 5:**
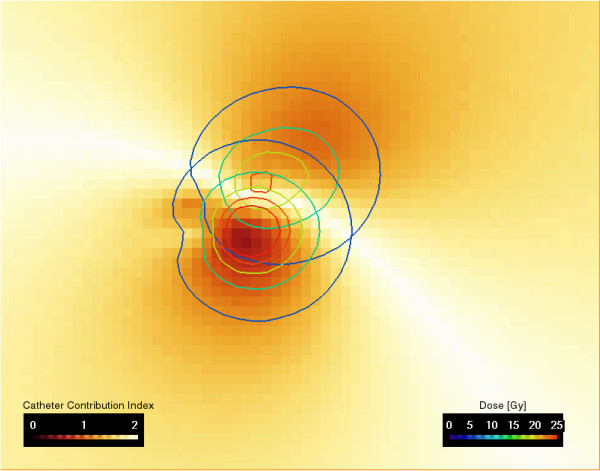
**Catheter contribution index**. The image showing the separated isodoses of two catheters for the patient in Fig. 1 and Fig. 4. The separated doses of the cranial and caudal catheter (Fig. 4) are used to calculate the catheter contribution index (Eq. 2) shown in color coding. In case of two equally contributing catheters, *D_i_*/*D_tot _*= 0.5 and *I_P _*= 2.0. *I_P _*is always in the range between 0 and 2.

The irradiation of a single voxel is prolonged as the number of dose-contributing catheters increases. Therefore, the catheter contribution index increases with the number of contributing catheters. In case of a single contributing catheter, *I_P _*= 0. In case of two equally contributing catheters, *D_i _*/*D_tot _*= 0.5, and *I_P _*= 2.0. *I_P _*is always in the range between 0 and 2. The separate treatment plans were combined in a voxelwise approach using an arithmetic module implemented in Amira 3.1, see Figure 5.

Catheter contribution index *I_P_*(*x*,*y*,*z*) was then averaged over the 3D maps of the mismatch areas, *I_P_*(*MA*+) and *I_P_*(*MA*-). We calculated an asymmetry coefficient with the following formula

(3) $$AC_{I}=\frac{I_{P}\left(MA+\right)-I_{P}\left(MA-\right)}{0.5\left(I_{P}\left(MA+\right)+I_{P}\left(MA-\right)\right)}$$

to compare the averaged catheter contribution indices *I_P_*(*MA*+) and *I_P_*(*MA*-) calculated using Eq. 2. A value of the asymmetry coefficient > 0 indicates that the catheter contribution index in „MA+" is higher than in „MA-", vice versa a value of the asymmetry coefficient < 0 indicates that the catheter contribution index in „MA+" is lower than in „MA-".

### Statistical analysis

The Generalized Estimating Equation (GEE) model was employed to statistically assess limits of catheter positioning accuracy and the effects of prolonged irradiation times on the tolerance dose of normal liver parenchyma. For a dataset consisting of repeated measurements (2 MRI sequences, 2 follow-up dates) of a variable of interest, a GEE model allows the correlation of outcomes within one individual to be estimated and taken into appropriate account in the equation which generates the regression coefficients and their standard errors [15,16]. The GEE model was calculated with SAS, Version 9.1 (SAS Institute Inc., Cary, NC, USA). A *p *< 0.05 was considered significant.

## 3 Results

The validation of image registration accuracy using landmarks yielded a mean deviation of 2.64 mm (25% quartile width (*Q*_25 _): 0.28 mm, 75% quartile width (*Q*_75_): 4.51 mm). Thus registration accuracy proved to be sufficient for evaluating catheter positioning accuracy. A total of 161 MRI examinations of 62 irradiation effects were performed 6 and 12 weeks after HDR-BT. Table 1 shows the mean volume and threshold dose of hepatocyte dysfunction (T1-w images) and interstitial edema (T2-w images) and corresponding liver tolerance doses as well as the standard deviation between the examinations at 6 and 12 weeks (6W and 12W).

**Table 1:** Normal liver tissue tolerance dose and volume of irradiation effect

	6w T1-w	12w T1-w	6w T2-w	12w T2-w
**n =**	**44**	**36**	**48**	**33**

Dose/Gy	13.7 ± 4.8	16.7 ± 5.0	14.3 ± 6.2	16.6 ± 6.4
Volume/cm^3^	190.3 ± 158.6	127.2 ± 118.8	190.0 ± 166.4	157.0 ± 143.5

Mean normal liver tissue tolerance dose and volume (± standard deviation) for interstitial edema assessed by hyperintensity on T2-w images and hepatocyte dysfunction assessed by hypointensity on T1-w images six/twelve weeks (6w and 12w) after HDR-BT (n: number of MRI examinations evaluated).

A total of 96 follow-up MRI examinations of 30 patients with 38 irradiation effects were assessed to analyze methodical limitations of catheter positioning accuracy. Only patients with unidirectionally implanted, i.e., nearly parallel, catheters were included in the evaluation. The median number of catheters inserted was 2 (***Q***_25_:1, ***Q***_75_: 3 catheters; range: 1-8 catheters).

Table 2 presents the axial, orthogonal, and total shifts (in mm) between the center coordinates of the irradiation effects and tolerance dose volumes in relation to the direction vectors of catheter implantation. The mean axial shift of hepatocyte dysfunction (T1-w images) was -5. 3 ± 5.4 mm and of interstitial edema (T2-w images) -5. 6 ± 6.0 mm in plane, indicating a shift of the irradiation effect volume against the corresponding tolerance dose volume in the direction of the catheter entry sites. The orthogonal shift as a surrogate for registration inaccuracy due to liver deformation was 4.0 ± 2.5 mm on T1-w images and 4.6 ± 2.6 mm on T2-w images.

**Table 2:** Shift between irradiation effect and planned dose distribution

	T1-w	T2-w
**n =**	**47**	**49**

Axial shift/mm	-5.3 ± 5.4	-5.6 ± 6.0
Orthogonal shift/mm	4.0 ± 2.5	4.6 ± 2.6
Total shift/mm	7.7 ± 4.4	8.4 ± 4.4
*AC_S_*	1.14 ± 0.43	1.04 ± 0.49

Mean axial, orthogonal, and total shift between center coordinates of the irradiation effect and planned dose distribution in relation to the direction vector of catheter implantation for T1-w and T2-w MRI data. Both follow-up dates, 6w and 12w, were evaluated together. A negative value of the axial shift indicates a shift into the direction of the catheter entry site. T1-w = hepatocyte dysfunction, T2-w = interstitial edema, n = number of MR examinations assessed.

The orthogonal and axial shifts between the center coordinates of the irradiation effect volume and the tolerance dose volume in relation to the direction vector of catheter implantation were highly correlated in the T1-w and T2-w MRI sequences (*p *= 0.003 and *p *< 0.001, respectively), as were the shifts between 6 and 12 weeks examinations (*p *= 0.001 and *p *= 0. 004, respectively).

The asymmetry coefficient of the orthogonal and axial shifts of the center coordinates of the irradiation effect and corresponding tolerance dose volume in relation to the direction vector of catheter implantation, *AC_S_*, was 1.14 ± 0.43 for hepatocyte dysfunction and 1.04 ± 0.49 for interstitial edema, indicating that the axial shift as a surrogate for catheter dislocation within the catheter track was predominant (*p *< 0.005). The asymmetry coefficient was significantly affected by the MRI sequence used (*p *= 0.014) but not by the change in the irradiation effect volume between the 6-week and 12-week examinations (*p *= 0.48).

A total of 129 follow-up MRI examinations of 44 patients with 48 irradiation effects were assessed to analyze the effect of prolonged irradiation time on the tolerance dose of normal liver parenchyma. All irradiation effects were induced by at least 2 brachytherapy catheters. The median number of catheters per irradiation effect was 4 (*Q*_25_: 3; *Q*_75_: 6 catheters; range: 2-11 catheters). The average time for complete application of the radiation dose was 1865 ± 758 seconds (range: 844 - 4432 seconds).

The volumes of the mismatch areas, „MA+" and „MA-", averaged over the 6-week and 12-week follow-up MRI examinations and T1-w and T2-w acquisitions, was 40.6 ± 28.9 cm^3 ^(23.5 ± 10.1%). The differences between the mismatch area volumes with regard to 6-week and 12 week follow-up examinations and T1-w and T2-w MRI are small, see Table 3. The average dose in „MA+" is approximately 12Gy 6 weeks and 14Gy 12 weeks after the intervention. The average dose in „MA-", is approximately 22-23Gy 6 weeks and 28Gy 12 weeks post intervention, see Table 3. The difference between the average doses in the mismatch areas is significant (*p *< 0.0001). The values for the catheter contribution indices in the mismatch areas, *I_P_*(*MA*+) and *I_P_*(*MA *-), as well as the asymmetry coefficients of the catheter contribution indices in the mismatch areas, *AC_I _*, with respect to hepatocyte dysfunction and interstitial edema and the corresponding follow-up dates are displayed in Table 3. The mean of *AC_I _*is > 0 in each subgroup, indicating that the catheter contribution index in „MA+" is slightly higher than in „MA-". *I_P_*(*MA*+) and *I*_*P*_(*MA*-) are significantly affected by the volume loss of the irradiation effect between the 6-week and 12-week follow-up examinations and consecutive shifts of the mismatch areas towards the high dose regions of the dose plan (*p *= 0.0014). There is no significant difference between *I_P_*(*MA*+) and *I_P_*(*MA*-) with respect to hepatocyte dysfunction and interstitial edema (*p *= 0.9).

**Table 3:** Mean dose, deviation of mean dose from normal liver tissue tolerance dose, and dose protraction in mismatch areas

	6W T1-w	12W T1-w	6W T2-w	12W T2-w
**n**	**35**	**27**	**40**	**27**

*D*(*MA*+)/Gy	12.0 ± 4.3	14.1 ± 4.4	11.8 ± 5.4	14.0 ± 6.3
*D*(*MA*-)/Gy	23.2 ± 11.9	28.5 ± 11.0	22.2 ± 11.6	27.7 ± 15.1
Δ*D*(*MA*+)/Gy	-2.1 ± 2.8	-3.2 ± 1.9	-2.1 ± 4.3	-3.0 ± 3.1
Δ*D*(*MA*-)/Gy	9.1 ± 7.5	11.2 ± 6.8	8.3 ± 6.6	10.7 ± 8.8
*I_P_*(*MA*+)	1.67 ± 0.33	1.69 ± 0.26	1.67 ± 0.31	1.70 ± 0.27
*I_P_*(*MA*-)	1.45 ± 0.39	1.35 ± 0.37	1.45 ± 0.37	1.39 ± 0.36
*AC_I_*	0.17 ± 0.28	0.25 ± 0.27	0.16 ± 0.26	0.23 ± 0.22
*V *(*MA *+/*MA*-)/cm^3^	42.0 ± 26.7	38.2 ± 31.2	40.8 ± 29.2	43.0 ± 33.1
*V *(*MA *+/*MA*-)/%	21.8 ± 11.1	23.9 ± 7.8	23.1 ± 0.8	27.0 ± 9.0

*D*(*MA*+), *D*(*MA*-): Average dose in mismatch areas; "*MA*+" for response at doses smaller than the tolerance dose and "*MA*-" for missing response at doses exceeding the tolerance dose.

Δ*D*(*MA*+), Δ*D*(*MA*-): Difference between the average dose in "*MA*+"and "*MA*-" and corresponding tolerance dose of the irradiation effect.

*I*_*P*_(*MA*+), *I*_*P*_(*MA*-): Catheter contribution index in "*MA*+" and "*MA*-".

*AC_I _*: Asymmetry coefficient between the catheter contribution indices in "*MA*+" and "*MA*-".

*V *(*MA *+/*MA*-): Volume of the mismatch areas "*MA*+" and "*MA*-" in percent and absolute value which is per definition identical for both areas.

Errors are given as standard deviation.

## 4 Discussion

In this study, we sought to assess two methodical aspects of HDR-BT: first, limits of catheter positioning accuracy and, second, effects of prolonged irradiation on the tolerance dose of normal liver parenchyma. The mean shift between the center coordinates of the irradiation effect volume and corresponding tolerance dose volume in relation to the direction vector of catheter implantation is ≈ - 5 mm in plane, indicating a shift of the irradiation effect in the direction of the catheter entry site. The shift is within the slice thickness of 5 mm of the treatment planning CT but larger than could be explained by registration inaccuracy, which is ≈ 3 mm, and inaccuracy due to local liver deformation in the follow-up images, resulting in an overall registration inaccuracy of ≈ 4-5 mm.

Determination of catheter positioning accuracy might be limited by the delineation of the brachytherapy catheters in the treatment planning CT since applicator geometry is entered manually. Partial volume effects in the treatment planning datasets could be a potential source of error in the treatment planning procedure, especially for catheters in oblique direction, since correct placement of the starting point of the catheter is dependent on conspicuity of the catheter tip.

Another limitation is the dislocation of catheters between acquisition of the planning CT and irradiation. Although the angiographic sheaths containing the catheters were secured to the skin by suture, retraction of the brachytherapy catheters within the catheter tracks might potentially occur due to patient movement, e.g., when the patient is transferred from the CT unit to the brachytherapy unit, and liver movement during respiration. However, the extent of the shift between an irradiation effect and the center of the planned dose distribution does not suggest a significant dislocation of the brachytherapy catheters within the catheter tracks.

The systematic shift between the irradiation effect volume and planned dose distribution has to be considered in treatment planning when defining the CTV to avoid underdosage of the tumor periphery. In our institution, the CTV comprises the tumor volume visible on contrast-enhanced CT scans plus a 5-mm safety margin. With regard to treatment planning, we conclude that a slice thickness exceeding 3 mm potentially impairs catheter positioning accuracy. We furthermore propose that it would be beneficial to increase the safety margin of the CTV in the direction of the catheter tips from 5 to 10 mm to avoid underdosage and consecutive recurrence at the tumor margin. The amount of mismatch (Table 3) between planned dose distribution and irradiation effect volume is determined by the registration accuracy or possibly by biological effects but does not allow to assess the reproducibility of the CTV. Two studies evaluated the accuracy of target positioning in extracranial stereotactic radiotherapy (ESRT) using special patient fixation. For mobile soft tissue targets, such as liver metastasis, Wulf et al. [17] reported mean target deviations of 0.9 ± 4.5 mm, 0. 9 ± 3.0 mm, and 3.4 ± 3.2 mm in the craniocaudal, anteroposterior, and lateral directions, respectively, when breathing control was applied. The mean 3D deviation of the targets was 6.1 ± 4.6 mm.

For single-fraction therapy, Herfarth et al. [18] reported mean target set-up deviations between treatment planning and treatment of 4. 0 ± 2.5 mm, 2.2 ± 1. 8 mm, and 2.2 ± 1.7 mm in the craniocaudal, anteroposterior, and lateral directions, respectively. The mean 3D deviation of the targets was 5.7 ± 2.5 mm.

The total in-plane deviation of the target location in our study was slightly higher, 4-6 ± 2-6 mm. However, we determined the effective positioning accuracy by comparing the shift between the irradiation effect in follow-up MRI and planned dose distribution. The authors quoted above compared treatment planning images with control CT datasets acquired before treatment [17,18] and did not evaluate the treatment effect.

Based on metric analysis of target mobility and set-up inaccuracy in the CT simulation prior to or during treatment, safety margins for defining the planning target volume (PTV) of about 5 mm in axial and 5 - 10 mm in craniocaudal direction are commonly added to the CTV in ESRT of lung and liver tumors [19]. In contrast to the present study, Wulf et al. evaluated the reproducibility of the CTV of lung and liver tumors within the planning target volume (PTV) over the entire course of hypofractionated treatment in CT simulation prior to application of each fraction [19]. The mean volume ratio of the PTV to the CTV was 2.2 ± 0.6 in liver targets. The authors showed that especially liver tumors with a CTV exceeding 100 cm^3 ^were susceptible to target deviation exceeding the standard safety margins for PTV definition. They suggested to increase the PTV by adding a larger safety margin to ensure adequate target dose deposition in these CTVs. In brachytherapy, the applicator moves to a certain extent together with the target and there is no need to increase the safety margin for larger tumors.

Catheter dislocation in brachytherapy was mainly investigated in fractionated HDR brachytherapy of the prostate, which differs from the technique used here in that a much larger number of catheters are implanted for more than one day. Imaging techniques (cone beam CT and CT) were used to assess catheter dislocation between the first and second fraction, i.e., over 24 hours. Foster et al. found a mean catheter displacement of 5. 1 mm, resulting in a significantly (*p *< 0.01) decreased mean prostate *V*_100 _(volume receiving 100Gy or more) from 93.8% to 76.2% [20]. Five patients had maximum catheter displacement exceeding 10 mm. Simnor et al. found a mean movement in caudal direction relative to the prostate base between the first and second fraction of 7. 9 mm (range 0-21 mm). Planning target volume dose *D*_90% _was reduced without movement correction by a mean of 27.8% [21]. Kim et al. found an average (range) magnitude of craniocaudal catheter displacement of 2.7 mm (- 6.0 to 13.5 mm) using bone markers and 5.4 mm (-3.75 to 18.0 mm) using the center of two gold markers [22]. Catheter dislocation in fractionated HDR brachytherapy of the prostate is in the same range as in the present study but, because of the much more complex irradiation geometry, the impact on dose coverage is much larger.

We assessed the effect of prolonged irradiation times on the tolerance dose of normal liver tissue to determine its relevance for treatment planning. A catheter contribution index served as a surrogate for prolonged pulsed dose administration in nonoverlapping areas of the irradiation effect volume and the corresponding tolerance dose volume. The catheter contribution index was slightly but significantly higher in „MA+" than in „MA-", indicating a prolongation of dose application in „MA+" compared to „MA-". Based on published data, we would have expected to find an increased tolerance dose of the liver parenchyma in areas irradiated for a longer time, i.e., by several catheters [6,7], even if the overall irradiation time is less than one hour [8]. However, we found a decreased tolerance dose of the liver parenchyma in areas where the radiation dose was applied by several catheters for a prolonged period of time.

We hypothesize that the effects of prolonged irradiation on the tolerance dose of normal liver tissue might have been obscured by other factors. For instance, biological effects such as reactive inflammatory changes may mimic irradiation effects, or scarring of the liver tissue induced by catheter insertion may cause retraction of the irradiation effect towards the catheter entry site. Furthermore, we propose that inaccuracies in the positioning of the brachytherapy catheters are more pronounced in areas where several catheters contribute to the total irradiation dose and that the total applied effective dose in „MA+" was higher than would have been expected from the treatment plan. Since steep dose gradients are an inherent quality of interstitial HDR-BT, the shift of active dwell positions of one or several catheters towards the tumor periphery would be sufficient to significantly increase the applied dose outside the CTV. As the number of catheters increases, the probability of a dose shift due to slight inaccuracy in catheter positioning likely increases as well.

We conclude that the effects of prolonged irradiation time are of minor importance for interstitial HDR-BT compared to other factors such as positioning accuracy of brachytherapy catheters and do not have to be taken into account in treatment planning in HDR-BT if the total irradiation time does not significantly exceed one hour.

The study has several limitations. Obviously one key issue of the study is the registration accuracy. The validation of registration accuracy was based on corresponding vessel bifurcations identified in the planning CT and follow-up MR images by an experienced radiologist [23,24]. We applied affine registration, allowing 12 degrees of freedom, which compensates for whole organ deformation and yielded an accuracy of ≈ 3 mm with respect to vessel bifurcations within the central parts of the liver, comparable to other studies [25,26]. Affine registration has been proven to be precise and robust for liver registration [25-27]. However, local liver deformation resulting from compression by adjacent organs (such as the stomach), different respiration levels, or the implanted catheters in the treatment planning CT data might not be sufficiently compensated for. To adequately compensate for these effects a finite element model-based deformable image registration would have been superior [23,24]. We tried to compensate for the limitations of affine registration by restricting the registration to the liver [25]. Using this procedure, we achieved a registration accuracy with a mean deviation of 2.64 mm, which was smaller than that of the nonrigid registration used by Elhawary et al. [28], for which the authors reported a mean target registration error of. 4.1 mm and a mean 95***^th^***-percentile Hausdorff distance of 3. 3 mm.

Second, the catheter contribution index has to be considered a rough simplification, merely providing a first estimate of the effect of prolonged dose administration. Dose administration was considered highly prolonged if the index was 2 (meaning that each catheter of the brachytherapy implant contributed < 50% of the irradiation dose in the mismatch area). It was considered fairly prolonged if the value was between 1 and 2 (indicating that more than 25% of the total irradiation dose in the mismatch area was applied by more than 1 catheter), and nonprolonged if the value was ≤ 1 (meaning that 75% or more of the total irradiation dose in the mismatch area was applied by 1 catheter only). Nevertheless, the tool is sufficient to rule out practically relevant effects of prolonged dose administration in HDR-BT in vivo.

## 5 Conclusions

In conclusion, positioning accuracy of brachytherapy catheters is sufficiently precise with approx. 5-6 mm. Accuracy was within the 5-mm slice thickness of the treatment planning CT. Thus positioning accuracy is potentially affected by inaccuracy in the delineation of the brachytherapy catheters during treatment planning due to partial volume effects in the planning CT. Retraction of the catheters within the catheter tracks during transfer of the patient from the CT unit to the brachytherapy unit might occur; however, this retraction is not pronounced. Therefore, CT-guided HDR-BT can be safely performed, even if CT and brachytherapy are not performed in the same unit. Effects of prolonged irradiation times on the tolerance dose of normal liver tissue are negligible compared to positioning accuracy of brachytherapy catheters and do not have to be taken into account in treatment planning if the total irradiation time does not significantly exceed one hour.

## 6 Competing interests

The authors declare that they have no competing interests.

## 7 Authors' contributions

LL, CW: data analysis, manuscript preparation.

PW, JR: study coordination, study design.

MS, KM: data acquisition.

SK: data analysis

All authors read and approved the final manuscript.
